# Influence of Salinity on Bacterioplankton Communities from the Brazilian Rain Forest to the Coastal Atlantic Ocean

**DOI:** 10.1371/journal.pone.0017789

**Published:** 2011-03-09

**Authors:** Cynthia B. Silveira, Ricardo P. Vieira, Alexander M. Cardoso, Rodolfo Paranhos, Rodolpho M. Albano, Orlando B. Martins

**Affiliations:** 1 Instituto de Bioquímica Médica, Universidade Federal do Rio de Janeiro, Rio de Janeiro, Brazil; 2 Instituto de Biologia, Universidade Federal do Rio de Janeiro, Rio de Janeiro, Brazil; 3 Instituto Nacional de Metrologia Normalização e Qualidade Industrial, Rio de Janeiro, Brazil; 4 Departamento de Bioquímica, Universidade do Estado do Rio de Janeiro, Rio de Janeiro, Brazil; Argonne National Laboratory, United States of America

## Abstract

**Background:**

Planktonic bacteria are recognized as important drivers of biogeochemical processes in all aquatic ecosystems, however, the taxa that make up these communities are poorly known. The aim of this study was to investigate bacterial communities in aquatic ecosystems at Ilha Grande, Rio de Janeiro, Brazil, a preserved insular environment of the Atlantic rain forest and how they correlate with a salinity gradient going from terrestrial aquatic habitats to the coastal Atlantic Ocean.

**Methodology/Principal Findings:**

We analyzed chemical and microbiological parameters of water samples and constructed 16S rRNA gene libraries of free living bacteria obtained at three marine (two coastal and one offshore) and three freshwater (water spring, river, and mangrove) environments. A total of 836 sequences were analyzed by MOTHUR, yielding 269 freshwater and 219 marine operational taxonomic units (OTUs) grouped at 97% stringency. Richness and diversity indexes indicated that freshwater environments were the most diverse, especially the water spring. The main bacterial group in freshwater environments was *Betaproteobacteria* (43.5%), whereas *Cyanobacteria* (30.5%), *Alphaproteobacteria* (25.5%), and *Gammaproteobacteria* (26.3%) dominated the marine ones. Venn diagram showed no overlap between marine and freshwater OTUs at 97% stringency. LIBSHUFF statistics and PCA analysis revealed marked differences between the freshwater and marine libraries suggesting the importance of salinity as a driver of community composition in this habitat. The phylogenetic analysis of marine and freshwater libraries showed that the differences in community composition are consistent.

**Conclusions/Significance:**

Our data supports the notion that a divergent evolutionary scenario is driving community composition in the studied habitats. This work also improves the comprehension of microbial community dynamics in tropical waters and how they are structured in relation to physicochemical parameters. Furthermore, this paper reveals for the first time the pristine bacterioplankton communities in a tropical island at the South Atlantic Ocean.

## Introduction

Microorganisms have large population sizes and show long-distance dispersal, high reproductive rates and remarkable genetic diversity, suggesting that they can cross environmental boundaries, including salinity, more frequently than multicellular organisms [1]. These particularities support the Baas-Becking hypothesis formulated in 1934, summed up as follows: “Everything is everywhere, but the environment selects” (revised by Hooper *et al.* [2]). Although this seems logical and plausible, the clustering test performed *in silico* by Lozupone and Knight [3] using annotated sequences from 202 globally distributed natural environments demonstrates that salinity is the major barrier to microbial communities, showing a strong environment-specific evolution between freshwater and marine bacteria.

Until the late 1980's, fresh and salt water planktonic bacteria were thought to be ecologically similar, despite minor differences such as some biotic interactions within the food web and sodium requirement. Salt-dependence in marine bacteria was not considered a fundamental ecological difference and species distribution and their physiology were thought to be similar to freshwater bacteria [4].

Since molecular methods started to be applied to the study of uncultivated microbial communities [5], [6], knowledge of microbial ecology in aquatic systems has been significantly increased [7]–[11]. The first difference seen in bacterial community composition in fresh and marine water was the dominance of *β-Proteobacteria* in the former, in contrast to the dominance of α- and γ- subdivisions of *Proteobacteria* in the latter [12]–[14]. Most bacterial sequences retrieved from freshwater environments were neither affiliated with known bacterial species nor with soil and marine relatives but clustered in a habitat-specific manner, leading to the conclusion that these were typical freshwater bacteria. Interestingly, this bacterial cluster presented a cosmopolitan distribution, including habitats located in different climatic zones [15].

Estuarine waters are dynamic environments due to the mixing of sediments, marine and freshwater, resulting in salinity and nutrient gradients. Shifts in physical, chemical, and microbiological properties between freshwater and adjacent coastal marine environments occur in short periods of time, driven by tides and freshwater flow, creating an intense abiotic pressure that influences the composition of bacterioplankton communities [16]. The presence and abundance of typical freshwater and marine bacterial taxa are closely related with these gradients and also with growth rates, viral lysis, predation, and retention times [17]–[20]. Long-term adaptability to different salinity conditions is also indicated by the ability of some organisms to occur in both marine and freshwater habitats [21]. In spite of a number of published studies of large estuaries and *in silico* comparisons between freshwater and seawater bacterioplankton, very few concerned South American tropical habitats.

The Atlantic rain forest, a species diversity hotspot [22]–[23], represents a substantial contribution of organic and inorganic material to the coastal waters of the Southwest Atlantic Ocean. Bacteria and fungi from Atlantic forest habitats have been analyzed mainly by culture-dependent methods [24]–[27]. By means of 16S rRNA gene libraries, it has been estimated that millions of new bacterial species exist in the Atlantic rain forest soil and phyllosphere [28]–[29]. As most of the Brazilian population lives in the coast, Atlantic forest habitats are greatly impacted by human activities. The Atlantic rain forest extends along the Brazilian coast from Rio Grande do Norte to Rio Grande do Sul states and has been reduced to less than 8% of its range [30]. The forest has a well-defined dry winter and rainy summers with high precipitation levels, with a mean annual rainfall of 1368 mm [31] that greatly increases river transport. This dynamic hydrology sustains a great biodiversity of flora and fauna which characterizes the Atlantic forest as a diversity hotspot [22]–[23].

**Figure 1:**
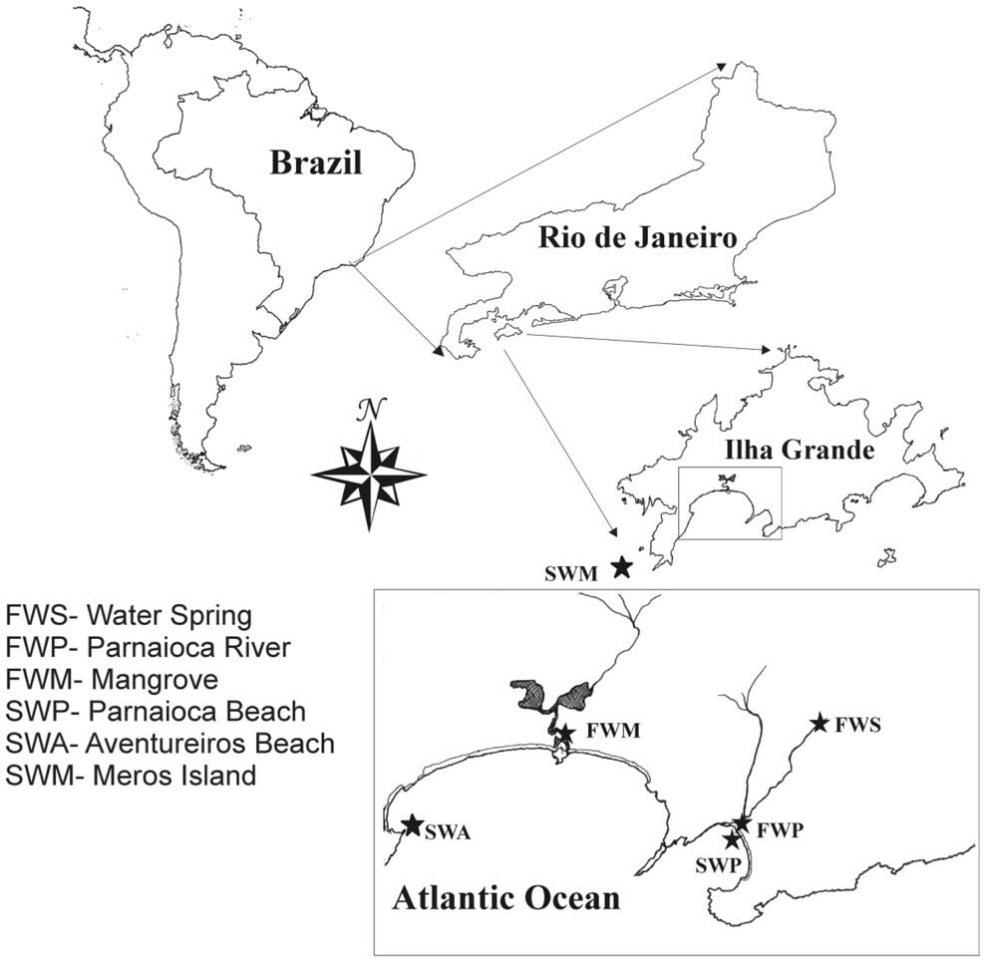
Map of the studied site and the six sampled locations. FWS – Parnaioca freshwater spring; FWP – Parnaioca river; FWM – mangrove; SWP – Parnaioca beach; SWA – Aventureiros beach; SWM – seawater near Meros island.

One of the few protected areas of the Atlantic rain forest is Ilha Grande island in Rio de Janeiro state, Brazil (Figure 1). Ilha Grande has some coastal marine and freshwater sites that may be considered as undisturbed. Based on the construction and analyses of 16S rRNA gene libraries, we compared bacterioplankton diversity in six representative habitats of Ilha Grande's aquatic ecosystems in the context of a salinity gradient. Here we present results that corroborate the idea of divergent evolution and the lack of transitions between marine and freshwater bacterial communities.

## Materials and Methods

### Sampling

The six analysed sites, three freshwater and three marine, are shown in Figure 1: FWS - a water spring (23°10′57.00″S/44°14′55.19″W); FWR - Parnaioca river (23°11′21.33″S/44°15′11.08″W); SWP - Parnaioca beach (23°11′24.77″S/44°15′15.07″W), just where Parnaioca river flows into; FWM - a mangrove (23°10′26.98″S/44°17′08.49″W) which, at the time of sampling, had the communication to the sea closed by a sand barrier; SWA - Aventureiros beach (23°11′24.53″S/44°18′58.06″W); SWM - two milles west from Ilha Grande island near Meros island (23°12′53.67″S/44°21′55.03″W). Water samples (5.8 Liters) were collected at 1 m depth (except for the water spring) on September 7, 2007 for DNA extraction and for abiotic and microbiological characterization (100 mL). Samples were kept on ice until processed in the laboratory.

### Chemical and microbiological parameters

Chemical data were determined in triplicates by standard oceanographic methods. Temperature, salinity, and pH were determined at the moment of sample collection using a field thermometer, a hand-held refractometer (Leica) and pH strips. Ammonia was measured by the indophenol method [32], nitrite by diazotation [33] and nitrate by reduction in a Cd-Cu column followed by diazotation [33]. Total phosphorus was evaluated by acid digestion to phosphate and silicate by reaction with molibdate [33].

Bacterial abundance was determined by flow cytometry [34] and bacterial production by ^3^H-leucine incorporation [35]–[37]. Specific production (SP) is an index calculated as the ratio Microbial Production versus Microbial Abundance [38] that allows comparisons of secondary productivity between environments with differences in prokaryotic counts.

### DNA extraction

The water samples were filtered through 0.2 µm Sterivex filters (Millipore, Bedford, MA, USA) after filtration through 3.0 µm to separate free-living microbes from larger organisms and particles. Total cellular nucleic acids were isolated by cell lysis with proteinase K and SDS, followed by phenol-chloroform extraction [39]. DNA integrity was checked on a 1% (w/v) agarose gel that was subsequently stained with Syber Green (FMC Bioproducts, Rockland, ME, USA) and the gel image was digitalized with Storm Image Scanner (GE Healthcare, Little Chalfont, UK).

### Bacterial 16S rRNA gene library construction

PCR was performed in 50 µl reaction mixtures (2.5 mM MgCl_2_, 0.2 mM deoxynucleoside triphosphates, 1 ng of each primer.µl^−1^, 2.5 U of High Fidelity *Taq* DNA polymerase [Promega], 1× PCR buffer and 200 ng of each environmental DNA sample, using the universal bacterial primers 27BF (5′-AGAGTTTGATCCTGGCTCAG-3′) [40] and 907RAB (5′-TTTGAGTTT MCTTAACTGCC-3′) [41]. PCR amplification began with a 5 min denaturing step at 94°C; this was followed by 25 cycles of 94°C for 90 seconds, 50°C for 90 seconds, and 72°C for 2 min. The final cycle was an extension at 72°C for 5 min. PCR products were concentrated and purified with a GFx PCR DNA and Gel Band Purification Kit (GE Healthcare) after electrophoresis on a 1% (w/v) agarose gel. PCR products were cloned into the pGEM-T cloning vector (Promega) and used to transform competent *E. coli* DH10B cells. Positive colonies for the blue-white colony screen used for this vector were picked and frozen at −70°C. Six 16S rRNA gene libraries were constructed from different environmental DNA samples.

### Sequence analyses and taxa identification

Approximately 192 clones from each clone library were submitted to sequence analysis. Plasmidial DNA from each clone (400 ng) was prepared and PCR-sequencing reactions with primer 27BF were carried out using the DYEnamic ET terminator cycle-sequencing kit (GE Healthcare). Partial 16S rRNA sequences were obtained by capillary electrophoresis on a MegaBace1000 DNA analysis system (GE Healthcare). Chromatograms were transformed into Fasta format with Phred software [42] and sequences with less than 300 bp and chimeras were removed prior to further analysis using MOTHUR. A total of 831 valid sequences with approximately 642 bp were compared with sequences in the Ribosomal Database Project II [43]. Sequences were also analyzed by BLAST [44] searches in GenBank database (http://www.ncbi.nlm.nih.gov) and were aligned with representative bacterial sequences obtained from the public databases using ClustalX software [45]. The partial 16S rRNA gene sequences generated in this study have been deposited in GenBank under accession numbers FJ717864-FJ718690. All submissions conform to the “Minimum information standards” recommended by the Genomic Standards Consortium [46].

### Biodiversity and phylogenetic analyses

Re-sampling and adjustment of the total number of sequence reads to identical sequencing depth was done before analysis [47]. Sequences were clustered as OTUs at an overlap identity cutoff of 97% or 80% by MOTHUR software [48]. Richness and diversity statistics including the nonparametric richness estimators ACE, Chao1 and the Shannon diversity index were calculated. The diversity of OTUs and community overlap were also examined using rarefaction analysis and Venn diagrams. Phylogenetic trees were constructed for marine and freshwater libraries with reference sequences from GenBank by the neighbor-joining algorithm based on distances calculated by the Kimura-2 method. This analysis was performed with the MEGA4 program [49] and bootstrap analysis with 1000 replications was used. Tree topology and distribution of hits along the tree were uploaded to the UniFrac computational platform [3], [50]. UniFrac is a beta diversity metric analysis that quantifies community similarity based on phylogenetic relatedness. In order to visualize distribution patterns of bacterial communities we used the UniFrac metric to perform PCA highlighted by significance. Libraries were sub-sampled randomly to test the consistency of the results.

### Statistical comparison between 16S rRNA libraries

In an attempt to determine the differences between clone libraries, we applied LIBSHUFF statistics [51] that uses Monte Carlo methods to generate homologous and heterologous coverage curves. Sequences were randomly shuffled 999 times between samples prior to the distance between the curves being calculated using the Cramér-von Mise statistic test. The DNADIST program of the PHYLIP package, using the Jukes-Cantor model for nucleotide substitution was used to generate the distance matrix analyzed by LIBSHUFF.

## Results

### Abiotic and microbiological parameters

Abiotic and microbiological parameters from each sampling site are shown in Table 1. Temperatures varied from 22 to 28°C. The low salinity found at Parnaioca beach (SWP) is explained by the input of freshwater from Parnaioca River to this site. In the same way, salinity in the mangrove (FWM) was typical of a freshwater environment due to strong rainfall that fell a few days before sampling which increased river input and blocked the communication of the mangrove with the sea by a sand barrier. For further analysis, the water spring, river and mangrove habitats were considered as freshwater environments, and Parnaioca, Aventureiros beach and Meros Island as marine environments. All are representative samples of the dynamic environmental conditions which characterize the Atlantic rain forest. Analysis of nitrogenated compounds showed the highest ammonia concentration at the mangrove site, FWM, while nitrate was the main compound in Parnaioca river, FWP. Nitrite concentrations ranged between 0.33 and 0.54 µM and silicate concentrations reached high values in the mangrove. Freshwater samples were more acidic than marine ones, with pH values ranging from 5.5 to 6.5 (Table 1).

**Table 1:** Abiotic and microbiological parameters.

	FRESHWATER			SEAWATER		
	FWS	FWP	FWM	SWP	SWA	SWM
a **Sal (S)**	0.09	0.83	0.73	26.67	33.64	32.63
b **T (°C)**	22	22	28	25	25	26
c **TP (µM)**	0.54	0.22	0.78	0.32	0.49	0.33
d **NH_3_ (µM)**	0.48	1.11	7.17	1.40	0.90	0.73
e **NO_2_^−^ (µM)**	0.44	0.33	0.54	0.39	0.38	0.41
f **NO_3_^−^ (µM)**	1.80	8.20	nd	0.95	0.90	0.73
g **SiO_2_ (µM)**	20.45	28.03	44.98	22.85	2.44	1.53
**pH**	5.5	5.5	6.5	7.0	7.5	7.0
h **MA (10^6^cells.mL^−1^)**	0.15	0.23	1.36	0.30	0.26	0.12
i **MP (µg C.L^−1^.h^−1^)**	0.26	1.97	3.44	1.08	0.76	0.44
j **SP (fg C.cell^−1^.h^−1^)**	1.69	8.51	2.53	3.54	2.88	3.43

a Sal, salinity;

b T, temperature;

c TP, total phosphorous;

d NH_3_, ammonia;

e NO_2_^−^, nitrite;

f NO_3_^−^, nitrate;

g SiO_2_, silicon;

h MA, microbial abundance;

i MP, microbial production; and

j SP, specific production.

**FWS** – Parnaioca freshwater spring; **FWP** – Parnaioca river; **FWM** – mangrove; **SWP** – Parnaioca beach; **SWA** – Aventureiros beach; **SWM** – seawater near Meros island.

Prokaryotic counts were in the range of 10^6^ cells per mL, being most abundant in the mangrove. Bacterial production values, which mean the heterotrophic activity, varied from 0.26 to 3.44 µg C.L^−1^.h^−1^. Although the highest heterotrophic activity was found in the mangrove, the bacterial production versus bacterial counts ratio (specific productivity - SP) was higher in the river. Marine samples presented SP values varying from 2.88 to 3.54 ag C.cell^−1^h^−1^ (Table 1).

### Clone library coverage, richness and diversity

The number of OTUs from each site as well as richness and diversity indexes calculated by MOTHUR [48] are shown in Table 2. The coverage of each library was calculated using the abundance-based coverage estimator (ACE). We also grouped freshwater (FWS, FWR, FWM) and marine sites (SWP, SWA, SWM) to perform these calculations. In order to account for uneven sampling efforts, the same number of sequences was randomly selected from each sample. The Parnaioca water spring, FWS, library had higher richness based on ACE, Chao1 and H′. Parnaioca river, the mangrove and Meros island libraries had the lowest richness values, but the H′ values were not far from the other libraries. Although no major differences among marine samples were found, SWP was the richest sample. Interestingly, the comparison between marine and freshwater libraries showed that, at 97% similarity level, bacterial richness and diversity of fresh and seawater communities are similar.

**Table 2:** Species richness estimates and diversity of 16S rRNA gene sequences as determined by MOTHUR software.

	FRESHWATER				SEAWATER			
	FW	FWS	FWP	FWM	SW	SWP	SWA	SWM
a **OTUs**	269	89	56	58	219	90	63	57
b **ACE**	2457	1024	184	101	762	233	187	296
**Chao1**	1018	564	130	85	543	220	134	252
c **H′**	5.33	4.43	3.72	3.83	5.07	4.42	3.90	3.69

a Number of unique OTUs defined by using the furthest neighbor algorithm in MOTHUR at 97% similarity.

b Abundance based coverage estimator (ACE).

c Shannon-weaver index of diversity (H′).

**FWS** – Parnaioca freshwater spring; **FWP** – Parnaioca river; **FWM** – mangrove; **SWP** – Parnaioca beach; **SWA** – Aventureiros beach; **SWM** – seawater near Meros island. **FW** and **SW** were calculated by merging the respective libraries.

**Figure 2:**
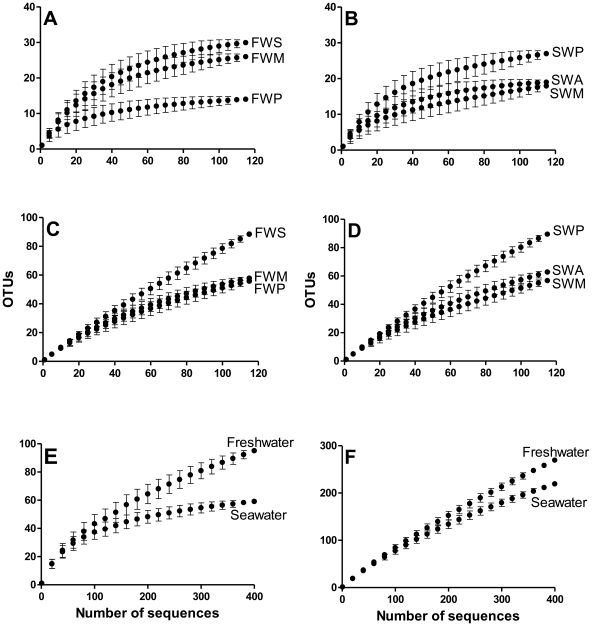
Rarefaction analysis of 16S rDNA clone libraries from Ilha Grande using a distance level of 80% (A, B and E) and 97% (C, D and F). In A and B or C and D each freshwater or marine water libraries are plotted, respectively. In E and F the three samples of seawater and the three samples of the freshwater were joined. FWS – Parnaioca freshwater spring; FWP – Parnaioca river; FWM – mangrove; SWP – Parnaioca beach; SWA – Aventureiros beach; SWM – seawater near Meros island.

All rarefaction curves at a high cutoff phylogeny resolution (97%) show that the diversity is very high and the total coverage of bacterial richness was not achieved. A decline in the rate of OTU detection at 80% cutoff indicates that the most dominant bacterial phyla have been detected for freshwater and marine samples. Rarefaction analysis at this cut-off revealed that freshwater environments were more diverse than marine ones, as well as at 97% cutoff (Figure 2). Additionally, Venn diagram shows that no OTUs are shared between fresh and marine water samples at species level (97%) indicating that the bacterial communities are completely different in these two kinds of environment.

### Bacterial Groups

**Figure 3:**
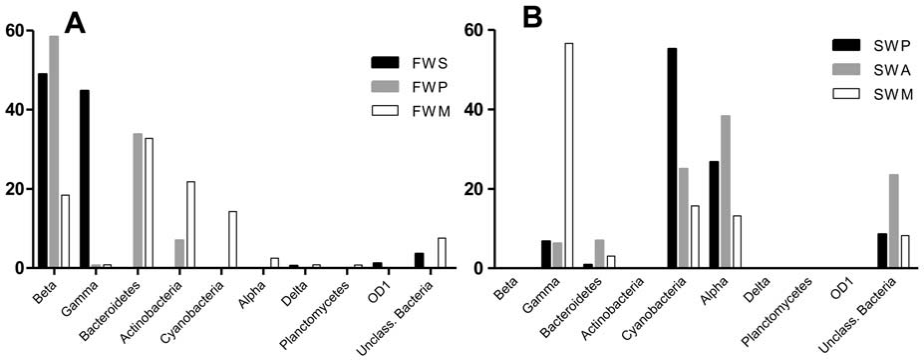
Distribution of sequences in bacterial phyla classified by the Classifier tool at RDP Database. Clones from freshwater libraries are shown in A and from seawater are shown in B. FWS – Parnaioca freshwater spring; FWP – Parnaioca river; FWM – mangrove; SWP – Parnaioca beach; SWA – Aventureiros beach; SWM – seawater near Meros island.

In order to reveal bacterial phyla composition in such diverse communities, sequences from each library were classified with the RPD classifier tool (http://rdp.cme.msu.edu/classifier). Marine samples showed a higher abundance of *Cyanobacteria*, *Alphaproteobacteria* while freshwater samples were dominated by *Betaproteobacteria* (Figure 3). *Gammaproteobacteria* were found mainly in the river (FWP) and Meros island (SWM) sites. A minor proportion of *Deltaproteobacteria* was observed in the FWP and mangrove (FWM) libraries. *Actinobacteria* were seen only in the river and mangrove environments, being more abundant in the latter one. *Bacteroidetes* were present in all the sites, except at the water spring. The newly described group OD1 was only found at the water spring and mangrove sites. A greater percentage of unclassified sequences were found in marine samples. Freshwater samples were richer at the phylum level than marine ones, with nine and four phyla represented, respectively.

### Phylogenetic Analysis

**Figure 4:**
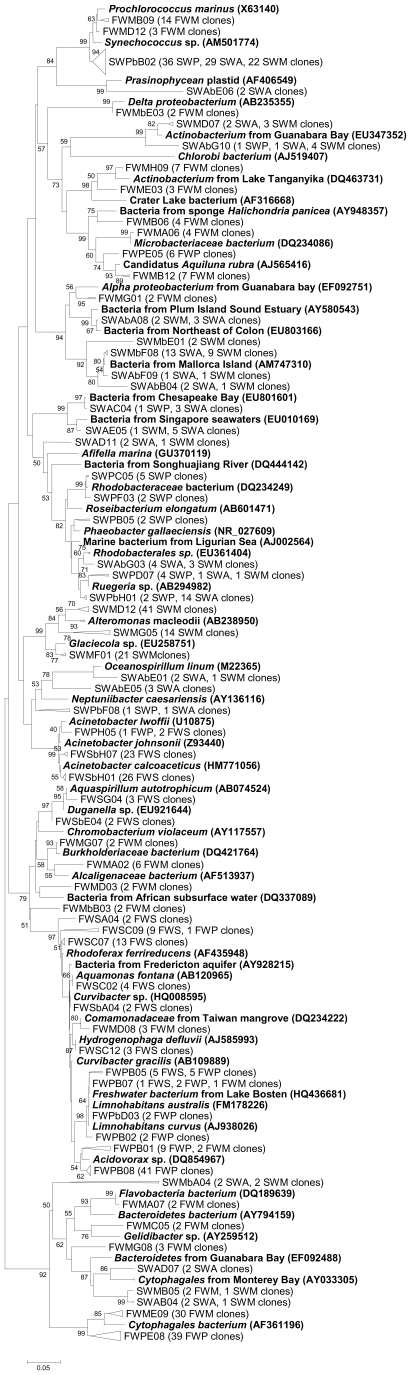
Phylogenetic tree of bacterial clones obtained in the freshwater or seawater locations. Reference sequences from GenBank (**in bold**). OTUs were defined by using a distance level of 3% by using the furthest neighbor algorithm in MOTHUR. One access number from each OTU is displayed. The tree topology is based on neighbor joining and bootstrap analysis was performed with 1000 replications. Bootstrap value >50 and representative OTUs are shown. More detailed trees can be found in Figures S1 and S2.

The phylogenetic tree allowed us to recognize the bacterial phylotypes that compose the groups listed above (Figure 4). The tree shows that most of our sequences were affiliated to environmental uncultured bacterial species. In freshwater samples, *Betaproteobacteria* sequences were affiliated to uncultured bacteria from lakes, freshwater ponds, aquifers, rivers, and subsurface freshwater. A great number of sequences from the river site were closely related to *Acidovorax* sp. The *Acinetobacter* was the most represented group among *Gammaproteobacteria*. Members of *Bacteroidetes* were not found in the water spring while they occurred in high percentage in the mangrove and river sites. Among all freshwater sequences, only two mangrove clones fell into the *Alphaproteobacteria* clade, being related to *Rhodobacteriaceae* retrieved from a Taiwan mangrove and river sediments, and two other OTUs fell into the *Deltaproteobacteria* group. At the mangrove, *Actinobacteria* were mainly represented by *Microbacteriaceae*. Additionally, in the mangrove and river libraries we found members of the recently proposed OD1 group, affiliated with a eutrophic lake bacterium. The *Cyanobacteria* found in the mangrove were related to marine species, different from those of the water spring site which were more related to drinking water system bacteria.

Phylogenetic analysis of the marine libraries revealed that *Cyanobacteria* were well represented by *Prochlorococcus* and *Synecchococus*, which is expected for coastal marine samples. Sequences from marine samples were mainly represented by *Alphaproteobacteria*. In this group, a representative clade with OTUs related to uncultured bacteria from Chesapeake Bay (USA), Mallorca Island (Spain), and Guanabara Bay (Brazil) and other clades with OTUs related to genera commonly found in marine waters, like *Roseobacter* and *Ruegeria*, were observed. The distribution of OTUs within *Gammaproteobacteria* followed this pattern, with a representative clade formed by uncultured bacteria from marine samples and by *Neptuniibacter* and *Oceanospirillum* species and another clade related to *Alteromonas*.

### Library Comparison

The comparison by LIBSHUFF statistics revealed that bacterial community composition differed significantly between marine and freshwater sampling sites. We obtained p<0.0001 for the comparisons of each marine library to each freshwater ones and also for the comparison of all marine sequences against all freshwater ones. Nevertheless, freshwater libraries were different among themselves whereas marine libraries were statistically similar (p = 0.0003 for the comparison between Parnaioca and Aventureiros, p = 0.0004 for Parnaioca and Meros, and p = 0.1718 for Aventureiros and Meros).

**Figure 5:**
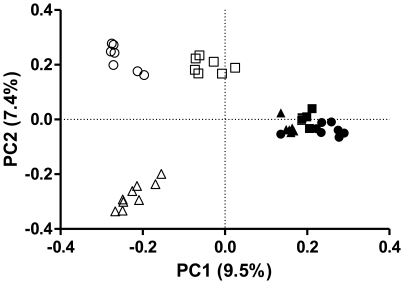
Match between bacterial communities in freshwater and seawater samples. Principal coordinates plots (PCA) were generated using the pair wise unweighted UniFrac distances. Freshwater in open symbols: FWS (**△**) – water spring; FWP (○) – Parnaioca river, FWM (□) - mangrove. Marine samples in filled symbols: SWP (•) – Parnaioca beach, SWA (▪) – Aventureiros beach, SWM (▴) – Meros island.

Through a scatter plot of the first two principal coordinates by the UniFrac analysis (Figure 5), PC1 and PC2 explained 9.5% and 7.4% of the data variation, respectively. The randomly constructed sub-libraries were grouped according to the original libraries. Marine libraries were separated from freshwater ones in the plot by PC1. The three marine libraries grouped together showing a high similarity with each other, whereas freshwater samples were dispersed in the plot and seem to be different among them. Additionally, the mangrove FWM clustered between freshwater and marine samples along the PC1 axis, which divides saline and other freshwater environments. This result corroborates the LIBSHUFF analysis, wherein only marine libraries reached high p values.

## Discussion

In this work we investigated for the first time the bacterioplankton diversity in the tropical island, Ilha Grande. This environment suffers very low anthropogenic impact and is located in the Brazilian coast at the South Atlantic Ocean. The differences found in community composition add new knowledge to planktonic bacteria distribution in freshwater and coastal marine ecosystems.

Many abiotic parameters, such as nutrient concentration and organic matter, are thought to influence the composition of natural bacterioplankton communities [52]–[53]. In the same manner, autochthonous biological activity can modify water chemical features [54]. In this study, nutrient concentrations in marine samples were similar to Sepetiba Bay values but lower than in the highly eutrophic Guanabara Bay [55], [39]. Both are economically important water bodies which lie geographically close to Ilha Grande. In the mangrove environment, high bacterial production contrasts with low specific productivity. A possible explanation is that many marine cells that entered into the mangrove are not active anymore because of the change in salinity. In opposition, the river community, that reached higher specific productivity values, seems to be a well-adapted community, which probably has a large supply of oxygen available for aerobic metabolism. In estuaries, shifts in bacterioplankton community composition along salinity gradients are related to residence and community doubling times [16], [18]. Specific productivity and bacterial abundance estimates allow microbial communities to be compared and can be used to measure the metabolic status of the planktonic microbes [38]. A particular estuarine community is formed in intermediate salinities when average metabolic status and, consequently, the doubling times are shorter than residence times. Although specific productivity values for Parnaioca river and all marine samples are around one order of magnitude higher when compared to a previous study in Guanabara bay, an urban, pollution impacted Brazilian bay [56], there is no water residence time as the river water flows directly into the sea without a transition area, causing an abrupt change in salinity, and giving no time for the development of local bacterial species. The consequence is a complete shift in community composition when Parnaioca river and Parnaioca beach are compared, despite the close proximity (50 m) of these two sites.

Typical marine clades, such as *Cyanobacteria* and the *Alpha* and *Gamma* subdivisions of *Proteobacteria* were more represented in marine coastal and open-sea samples, not just in our data but also in the literature [16], [57]. However, in contrast to previous studies that found a low relative abundance of phototrophic *Cyanobacteria* compared to heterotrophic bacteria [58]–[59], members of *Synechococcus* and *Prochlorococcus* were one of the most abundant groups in Ilha Grande marine samples.

The most abundant group in water spring, river, and mangrove sites were the *Betaproteobacteria*, a typical freshwater clade [12] that was not recorded in marine samples. Recovery of 16S rRNA gene clones affiliated to *Betaproteobacteria* is common in libraries constructed from coastal samples, but few to no *Betaproteobacteria* have been reported by open ocean surveys [16], [57], [60]–[62]. These findings lead to the idea that bacterioplankton represented by these lineages have a probable freshwater origin and are adapted to coastal marine environments and could be representative of bacterioplanckton phylotypes that transit between freshwater and marine habitats [63]. However, the present data clearly do not support this proposal, since no *Betaproteobacteria* was retrieved from our marine libraries.

The *Gammaproteobacteria* and *Bacteroidetes* clades were well represented in both saline and freshwater environments. This might be a consequence of the presence of closely related marine phylotypes of common freshwater taxa [59]. In fact, the bacterial phylotypes belonging to these two clades encompass distantly related organisms in freshwater and marine samples, as seen in the phylogenetic trees, indicating an evolutionary separation between these marine and freshwater lineages [1]. In the marine sites, several *Gammaproteobacteria* and *Bacteroidetes* related OTUs were affiliated to sequences from marine habitats of different geographic areas, indicating that these are worldwide distributed bacteria.

Our data show a strong spatial heterogeneity of bacterial community composition in Ilha Grande. Most libraries, except when the three marine libraries are compared among themselves, are statistically different to each other. This most likely reflects the remarkable abiotic differences of these environments, especially salinity. This was also observed by Vieira et al [56] in Guanabara Bay, but contrasts to the results found for Chesapeake Bay (USA), where only temporal variation was significant [64]. The water spring is an interesting case, as it is highly different from the other environments, including other freshwater habitats. This may be explained by a strong influence of soil, plant-associated and underground water bacterial communities.

As seen by Lozupone [3], our data showed a clear separation between freshwater and marine libraries. The PC1 axis represented the saline barrier which segregates marine and freshwater bacterial communities. In fact, salinity is pointed out as the major environmental determinant of aquatic microbial community composition, rather than extremes of temperature, pH, or other physical and chemical factors by the global pattern of the bacterial diversity [3]. Recently, deep evolutionary divergence between marine and freshwater SAR11 lineages was seen not only by means of 16S phylogenetic constructions and Unifrac analysis, but also by Fragment Recruitment Analysis using metagenomic libraries from environments of different salinities [65]. Although our marine samples clustered together in the PCA analysis, freshwater ones were dispersed in the plot, showing a higher heterogeneity among these environments. Interestingly, mangrove communities cluster along the PC1 axis, between saline and other freshwater environments. This could be a result of the recent changes in salinity due to a sand barrier formation and the intense rainfall that brought a large input of freshwater to this habitat. The dispersion seen among the freshwater environments has been observed in other studies [3], [65] and is probably the result of complex interactions between biotic and abiotic factors, not only salinity, which ultimately shape communities in natural habitats.

Community composition changes across salinity gradients probably lead to changes in expression patterns that can modify the way in which organisms interact with each other and with the environment. In fact, seasonal changes in bacterial gene expression patterns across the salinity gradient in the Columbia river was recently observed by microarrays [66].

In summary, our results support the notion of ecologically defined bacterial species and processes and increase our knowledge about the relationships between bacterial diversity and environmental parameters in a tropical region.

## Supporting Information

Figure S1 **Phylogenetic tree of bacterial clones obtained in the freshwater locations.** Reference sequences from GenBank (**in bold**). OTUs were defined by using a distance level of 3% by using the furthest neighbor algorithm in MOTHUR. The tree topology is based on neighbor joining and bootstrap analysis was performed with 1000 replications. Bootstrap value <50 and singletons are not shown. FWS (**△**) – Parnaioca freshwater spring; FWP (○) – Parnaioca river; FWM (□) – mangrove.
(TIF)

Figure S2 **Phylogenetic tree of bacterial clones obtained in seawater locations.** Reference sequences from GenBank (**in bold**). OTUs were defined by using a distance level of 3% by using the furthest neighbor algorithm in MOTHUR. The tree topology is based on neighbor joining and bootstrap analysis was performed with 1000 replications. Bootstrap value <50 and singletons are not shown. SWP (•) – Parnaioca beach; SWA (▪) – Aventureiros beach; SWM (▴) – seawater near Meros island.
(TIF)
