# Supplementary figures and images for: Influence of Salinity on Bacterioplankton Communities from the Brazilian Rain Forest to the Coastal Atlantic Ocean
Source: PLoS One. 2011 Mar 9;6(3):e17789. doi: 10.1371/journal.pone.0017789 (PMC3052384; doi:10.1371/journal.pone.0017789)

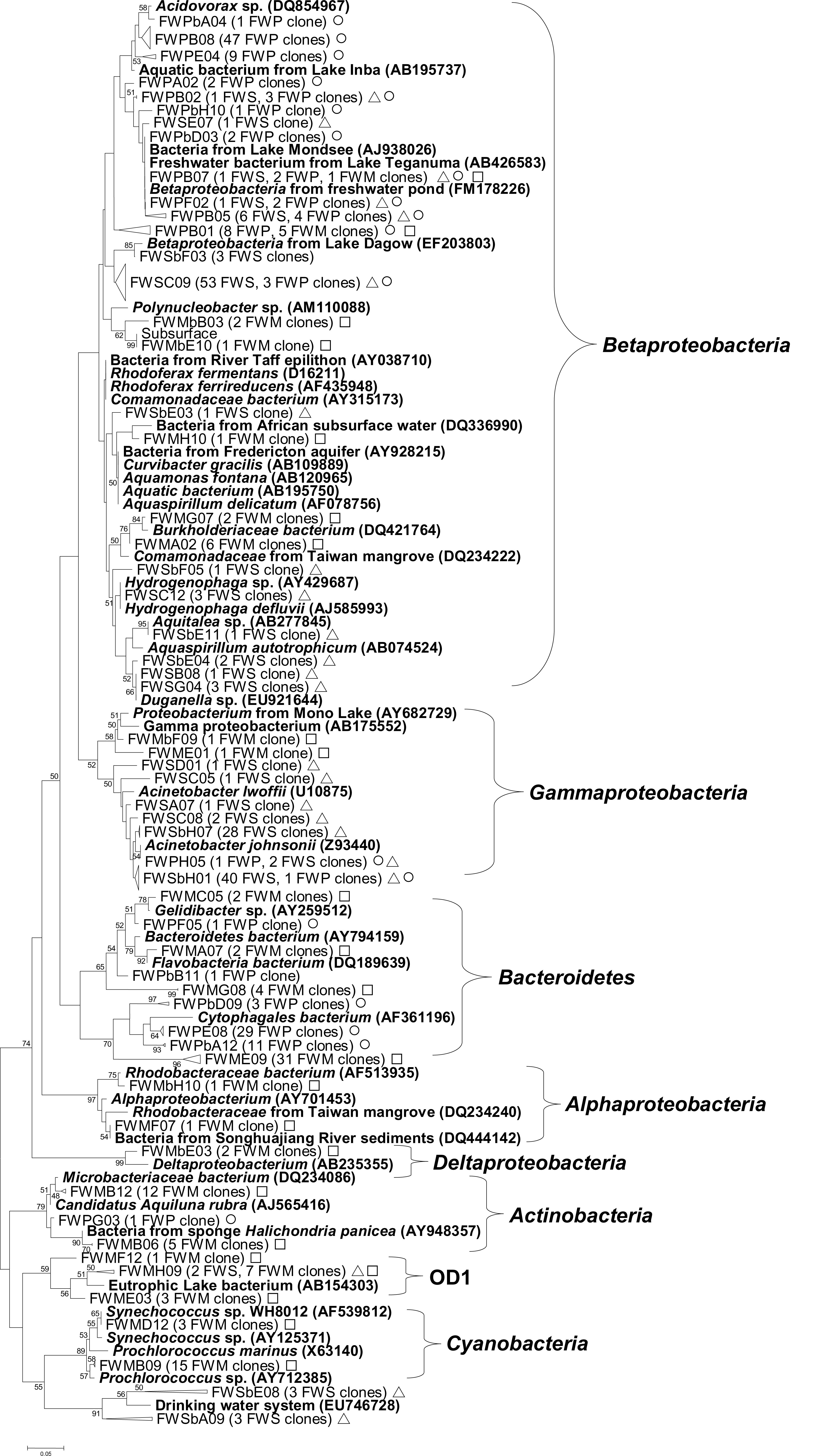

Supplement: Figure S1 — Phylogenetic tree of bacterial clones obtained in the freshwater locations. Reference sequences from GenBank (in bold). OTUs were defined by using a distance level of 3% by using the furthest neighbor algorithm in MOTHUR. The tree topology is based on neighbor joining and bootstrap analysis was performed with 1000 replications. Bootstrap value <50 and singletons are not shown. FWS (△) – Parnaioca freshwater spring; FWP (○) – Parnaioca river; FWM (□) – mangrove. (TIF) [file pone.0017789.s001.tif]

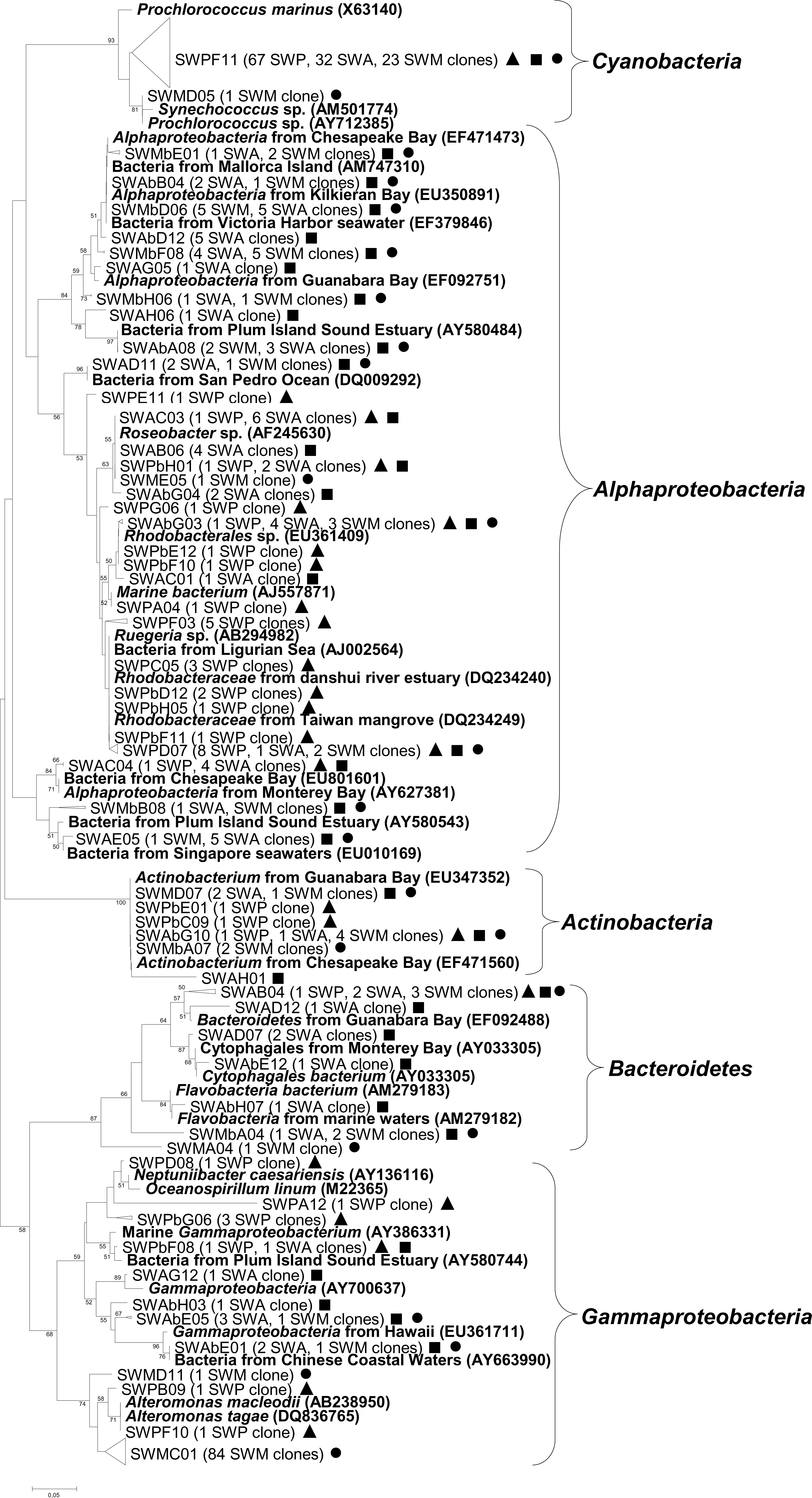

Supplement: Figure S2 — Phylogenetic tree of bacterial clones obtained in seawater locations. Reference sequences from GenBank (in bold). OTUs were defined by using a distance level of 3% by using the furthest neighbor algorithm in MOTHUR. The tree topology is based on neighbor joining and bootstrap analysis was performed with 1000 replications. Bootstrap value <50 and singletons are not shown. SWP (•) – Parnaioca beach; SWA (▪) – Aventureiros beach; SWM (▴) – seawater near Meros island. (TIF) [file pone.0017789.s002.tif]
